# Cost-effectiveness and public health impact of typhoid conjugate vaccine introduction strategies in Bangladesh

**DOI:** 10.1016/j.vaccine.2024.03.035

**Published:** 2024-04-19

**Authors:** Christopher Weyant, Yogesh Hooda, Sira Jam Munira, Nathan C. Lo, Theresa Ryckman, Arif M. Tanmoy, Naito Kanon, Jessica C. Seidman, Denise Garrett, Samir K. Saha, Jeremy D. Goldhaber-Fiebert, Senjuti Saha, Jason R. Andrews

**Affiliations:** a Department of Health Policy and Center for Health Policy, Stanford School of Medicine and Freeman Spogli Institute, Stanford University, Stanford, CA, United States; b Child Health Research Foundation, Dhaka, Bangladesh; c Division of Infectious Diseases and Geographic Medicine, Department of Medicine, Stanford University, Stanford, CA, United States; d Johns Hopkins Bloomberg School of Public Health, Johns Hopkins University, Baltimore, MD, United States; e Sabin Vaccine Institute, Washington, DC, United States; f Department of Microbiology, Bangladesh Shishu Hospital and Institute, Dhaka, Bangladesh

**Keywords:** Typhoid, Enteric fever, Vaccines, Bangladesh, Cost-effectiveness, Model, Seroincidence

## Abstract

**Purpose:**

Typhoid fever causes substantial morbidity and mortality in Bangladesh. The government of Bangladesh plans to introduce typhoid conjugate vaccines (TCV) in its expanded program on immunization (EPI) schedule. However, the optimal introduction strategy in addition to the costs and benefits of such a program are unclear.

**Methods:**

We extended an existing mathematical model of typhoid transmission to integrate cost data, clinical incidence data, and recently conducted serosurveys in urban, semi-urban, and rural areas. In our primary analysis, we evaluated the status quo (i.e., no vaccination) and eight vaccine introduction strategies including routine and 1-time campaign strategies, which differed by age groups targeted and geographic focus. Model outcomes included clinical incidence, seroincidence, deaths, costs, disability-adjusted life years (DALYs), and incremental cost-effectiveness ratios (ICERs) for each strategy. We adopted a societal perspective, 10-year model time horizon, and 3 % annual discount rate. We performed probabilistic, one-way, and scenario sensitivity analyses including adopting a healthcare perspective and alternate model time horizons.

**Results:**

We projected that all TCV strategies would be cost saving compared to the status quo. The preferred strategy was a nationwide introduction of TCV at 9–12 months of age with a single catch-up campaign for children ages 1–15, which was cost saving compared to all other strategies and the status quo. In the 10 years following implementation, we projected this strategy would avert 3.77 million cases (95 % CrI: 2.60 – 5.18), 11.31 thousand deaths (95 % CrI: 3.77 – 23.60), and save $172.35 million (95 % CrI: −14.29 – 460.59) compared to the status quo. Our findings were broadly robust to changes in parameter values and willingness-to-pay thresholds.

**Conclusions:**

We projected that nationwide TCV introduction with a catch-up campaign would substantially reduce typhoid incidence and very likely be cost saving in Bangladesh.

## 1 Introduction

Typhoid fever is an acute febrile illness caused by *Salmonella enterica* subspecies *enterica* serovar Typhi (*S.* Typhi) [1], [2]. This bacterium is transmitted via the fecal-oral route, primarily through contaminated water and food. While symptoms of infection are often mild, severe cases can be life threatening due to complications such as intestinal perforation, gastrointestinal bleeding, multi-organ failure, and septic shock. There are an estimated 12 to 24 million cases of typhoid fever each year, largely concentrated in impoverished areas of Asia and Africa [1].

Bangladesh is one of the top five countries in terms of highest per capita typhoid fever clinical incidence, deaths, and disability-adjusted life years (DALYs), according to the Global Burden of Disease (GBD) [3]. Further, *S.* Typhi antimicrobial resistance is increasing in the country. More than 95 % of *S.* Typhi isolates are non-susceptible to fluoroquinolones, and azithromycin resistance has recently emerged among multiple lineages [4], [5]. In terms of the epidemiology of typhoid in Bangladesh, people living in poor communities lacking clean water, food, and sanitation generally have high clinical incidence [6]. Children also have high clinical incidence [6]. The clinical incidence is currently only known for Dhaka, which is Bangladesh’s capital and largest city; however, it is thought to be substantially lower in less densely populated areas. The primary strategies for addressing typhoid in Bangladesh have been through improvements in water, sanitation, and hygiene (WASH) [6], though these measures have not been sufficient to prevent or contain the spread of typhoidal *Salmonellae*.

Traditional typhoid vaccines such as the live-attenuated Ty21a and Vi polysaccharide vaccines have several critical shortcomings that limited their adoption in routine immunization programs [7], [8], [9]. They have modest efficacy, offer short-term protection, and are not approved for use in young children, which prevents them from being included in routine vaccination programs such as the Expanded Program on Immunization (EPI). None of these typhoid vaccines were substantively used in Bangladesh in the public or private sector. In contrast, new typhoid conjugate vaccines (TCVs) have been found to confer a greater degree of protection and generate more durable immune responses [10], [11], [12], [13], [14], [15]. They can also be administered to children as young as six months of age, which enables them to be included in routine childhood vaccination programs [16]. There are currently no paratyphoid vaccines; however, paratyphoid fever accounts for ∼ 12 % of enteric fever cases in Bangladesh [17]. A wide variety of vaccination programs have been successfully implemented in Bangladesh [18]. Relatively recent examples include a measles-rubella catch-up campaign (2014), introduction of pneumococcal conjugate vaccine and IPV (2015), and a demonstration of the human papillomavirus (HPV) vaccine in school-aged girls in Gazipur (2016).

The government of Bangladesh plans to introduce TCVs in its EPI schedule; however, there remain critical questions about optimal introduction strategies, including age groups, geographic focus, and the inclusion of catch-up campaigns. Further, the anticipated impact of TCV introduction on cases and deaths, as well as expenditures and costs averted, is unclear. To address these gaps, we extend an existing typhoid model [19], leveraging clinical incidence and cost data, as well as newly conducted serosurveys in urban, semi-urban, and rural areas. We integrate these data sources to project the clinical impact and cost-effectiveness of TCV introduction strategies in Bangladesh.

## 2 Materials and methods

### 2.1 Overview

We extended an existing mathematical model of typhoid transmission [19] and evaluated various strategies for typhoid conjugate vaccination in Bangladesh. Our extended model is a dynamic transmission compartmental model programmed in Julia [20]. We calibrated the model to empirical data on both clinical incidence and seroincidence in Bangladesh, and we then projected outcomes over the next 10 years for each strategy including clinical incidence, seroincidence, deaths, costs, disability-adjusted life years (DALYs), and incremental cost-effectiveness ratios (ICERs). The seroincidence rate is equal to the clinical plus subclinical incidence rates. We also performed probabilistic, one-way, and scenario sensitivity analyses.

### 2.2 Model

**Fig. 1:**
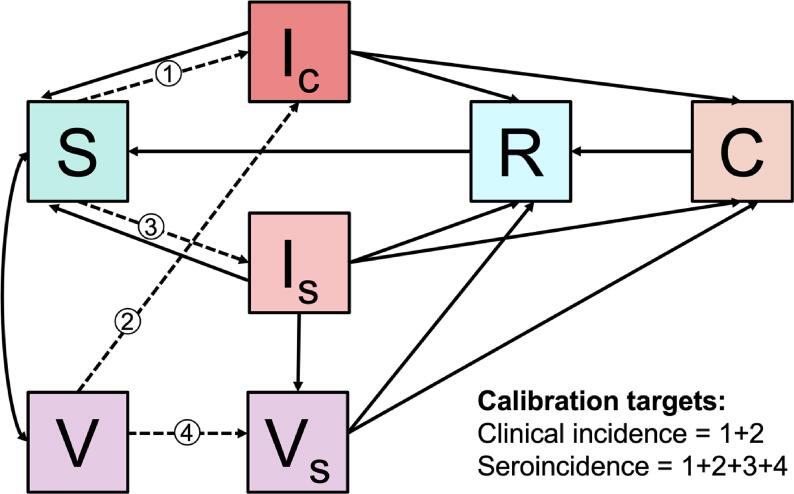
Model schematic. The model stratifies the population of Bangladesh by typhoid status, Dhaka and non-Dhaka residence, and age group. The model considers seven mutually exclusive and collectively exhaustive typhoid statuses including susceptible (S), clinically infected (I_c_), subclinically infected (I_s_), recovered (R), carrier (C), vaccinated (V), and vaccinated with subclinical infection (V_s_). Arrows show possible transitions between typhoid statuses; dashed arrows are numbered and show transitions corresponding to new infections. In addition to transitions between typhoid statuses, the model allows for births, aging, mortality (background and typhoid-specific), and migration between Dhaka and non-Dhaka. The model is calibrated to empirical data on typhoid clinical incidence and seroincidence in Bangladesh.

Our extended model is a dynamic transmission compartmental model (Fig. 1). The model stratifies the population of Bangladesh by typhoid status, Dhaka and non-Dhaka residence, and age group. The mutually exclusive and collectively exhaustive typhoid states include susceptible, clinically infected, subclinically infected, recovered, carrier, vaccinated, and vaccinated with subclinical infection. Subclinical infections contribute to transmission but are less infectious than clinical infections. The age groups include 0 to < 9 months, 9 to < 14 months, 14 to < 24 months, 2 to < 5 years, 12 age groups from 5 to < 65 years each spanning 5 years, and 65 + years. There are separate age groups for 9 to < 14 months and 14 to < 24 months as this allowed us to perform a scenario analysis in which routine TCV doses are administered with the second dose of the measles vaccine instead of the first. We stratified the population by Dhaka and non-Dhaka residence as the seroincidence data for the populations sampled outside of Dhaka were similar but distinct from the seroincidence data for the population sampled inside Dhaka. In addition to changes between the typhoid states, the model allows for changes corresponding to births, aging, mortality (background and typhoid-specific), and migration between Dhaka and non-Dhaka. There is not mixing between Dhaka and non-Dhaka in terms of the force of infection. See Text S1 for a detailed model description.

We calibrated 8 model parameters governing transmission to empirical data on clinical incidence and seroincidence for Dhaka in addition to seroincidence for non-Dhaka. Clinical incidence data for non-Dhaka was not available. These parameters included the typhoid transmission rates for 4 age groups (0–4, 5–14, 15–29, and 30 + years), which were assumed to account for both short-cycle (person-to-person) and long-cycle (water-borne) transmission. The two transmission routes were not modeled separately due to identifiability concerns, which is common in typhoid models [21]. Other calibrated parameters include the fraction of clinical versus subclinical infections, rate of loss of immunity, and fractions of immune response after clinical infection and subclinical infection, respectively (i.e., the proportions of people who move to the recovered versus susceptible state following infection). Of the calibrated parameters, only the beta parameters were allowed to vary between Dhaka and non-Dhaka. Due to the number of parameters in the model, computational complexity, and identifiability issues, we did not calibrate all parameters. For those with good literature estimates, we sampled 1,000 times from distributions in the literature. For those that are more setting specific and/or could not be derived from the literature, we calibrated. To calibrate the model, we conducted directed search for the calibrated parameters using Nelder-Mead with Poisson likelihood-based goodness of fit. This allowed us to determine transmission parameters that, when used in the model, generate clinical incidence and seroincidence estimates that are consistent with those empirically observed.

The model projects various outcomes. Model outcomes include clinical incidence, seroincidence, deaths, costs, DALYs, and ICERs for each vaccination strategy. For costs, we adopted a societal perspective and included medical and nonmedical vaccination costs, costs of typhoid illness, and productivity costs. As we consider costs from a societal perspective, the payer is not explicitly considered; however, Gavi support is often critical to governments’ decisions concerning vaccine introduction. We did not include Gavi support in the scenario analysis from the healthcare sector perspective as the payer is likewise immaterial. In the base case analysis, we considered a model period of 10 years and recorded outcomes monthly. We discounted both DALYs and costs at a 3 % annual discount rate as is typical in cost-effectiveness analyses [22].

### 2.3 Data

We parametrized the model for Bangladesh. Table 1 provides an overview of the data sources. We obtained clinical incidence for Dhaka from the Surveillance for Enteric Fever in Asia Project (SEAP) [17], [23]. We obtained seroincidence estimates from community and school-based serosurveys conducted as part of the Seroepidemiology and Environmental Surveillance (SEES) study. These serosurveys were performed in 7 communities that included urban, semi-urban, and rural areas, with population densities ranging from 782 to 71,347 people/km^2^ (Text S2, Tables S1 and S2). Seroincidence was estimated using published methods [23]. We adjusted seroincidence to reflect typhoid seroincidence as opposed to typhoid and paratyphoid seroincidence using data from SEAP for Dhaka [17]. This adjustment assumes that the prevalence ratio between typhoid and paratyphoid is the same in Dhaka as in other parts of Bangladesh. Since one of the communities with serosurvey data was Dhaka, we used those seroincidence values for Dhaka. For non-Dhaka, we estimated the seroincidence by first fitting a log-linear model of seroincidence versus catchment population density with data from the non-Dhaka communities. We estimated catchment population density by using population density data for Bangladesh at a resolution of 100 m from WorldPop [24]. We then projected the seroincidence and associated uncertainty for non-Dhaka using an estimate of the population density of non-Dhaka calculated with data from the World Bank [25]. WorldPop provides population data at a resolution of 100 m. We used these data to estimate catchment population density as this resolution was necessary and not available from other sources. We used population data from the World Bank for other purposes when we did not need the finer resolution. Both of these datasets are based on and consistent with census data. The dataset from WorldPop that we use (the top-down dataset) maintains the census estimates but essentially also estimates how the population is distributed at a finer scale.

**Table 1:** Model parameters.

**Parameter**	**Mean**	**Source**
**Transmission and Natural History**		
Transmission rate	Varies by age group and setting	Calibrated (see Supplement)
Symptomatic infections (% of total)	4.26 % [3.37–5.16]	Calibrated (see Supplement)
Duration of immunity against clinical infection	19.18 years [13.34–29.19]	Calibrated (see Supplement)
Percent of clinical infections that mount protective immune response (i.e., transition from infected to recovered)	60.70 % [37.59–74.84]	Calibrated (see Supplement)
Percent of subclinical infections that mount protective immune response (i.e., transition from infected to recovered)	29.19 % [25.00–37.32]	Calibrated (see Supplement)
Case fatality fraction	0.30 % [0.05–0.55]	*Yu et al.*[46]
Duration of infection	29.54 days [5.21–91.55]	*Mejia et al.*[36], assume shedding lasts twice as long as symptoms based on data from challenge studies
Relative infectiousness of sub-clinical infections	72 % [44–100]	Control group data from *Darton et al.*[47]
Relative infectiousness of vaccinated sub-clinical infections versus non-vaccinated sub-clinical infections	64 % [58–71]	*Gibani et al.*[27]
**Carrier Epidemiology**		
Percent of infections that progress to carrier	Varies by age	Calculated based on Woodward unpublished report, reported in *Gibani et al.*[27]; *Ames et al.*[48]
Duration of carriage	10 years [5–15]	Based on *Ames et al.*[48]*, Bhan et al.*[49]*, Gunn et al.*[50]
Relative infectiousness of carriers, compared with acute infections	7.5 % [5.5–9.5]	Calibrated estimate from *Lo et al.*[41]
**Vaccine Characteristics**		
Vaccine efficacy	85 % [76–91]	*Qadri et al.*[15]
Duration of immunity (TCV)	20 years [15–30]	Calculations from seroconversion data from *Mai et al.*[51] and Bharat Biotech (unpublished)
**Vaccine Coverage**		
Routine coverage	98 % [96–99]	UNICEF [52]
Campaign coverage	79 % [62–92]	Based on vitamin A from DHS [53]
School coverage	79 % [62–92]	Based on vitamin A from DHS [53]
Status quo coverage	0 %	Assumed
**Vaccine Costs per Dose**		
Vaccine	$1.00	Price announcements from the manufacturer [28]
Syringes and safety equipment	$0.25 [0.24–0.27]	*Portnoy et al.*[54]
Routine delivery – total costs	$0.89 [0.83–0.95]	*Pecenka et al.*[29]
Campaign delivery – total costs	$1.04 [0.94–1.15]	*Sarker et al.*[30]
School-based delivery – healthcare costs	$1.23 [0.61–1.85]	Literature review of other school-based vaccination delivery costs [31], [32], [33], [34]
School-based delivery – start-up healthcare costs	$2.12 [1.60–2.64]	Literature review of other school-based vaccination delivery costs [31], [32], [33], [34]
School delivery – out of pocket and time costs	$0	No incremental costs because delivery is to children who are already in school
**Cost of Illness (for symptomatic infections)**		
Costs of illness – adult	$165.61 [79.04–288.75]	Calculated from Mejia *et al.*[35]*,* Mejia *et al.*[36]*,* Mejia *et al.*[37]
Costs of illness – pediatric	$75.66 [33.87–137.97]	
**Quality of Life and Disability**		
Severe cases (% of symptomatic infections)	27 % [26–28]	*Longley et al.*[55]
Moderate cases (% of symptomatic infections)	73 % [72–74]	*Longley et al.*[55]
Ileal perforation (% of severe cases)	0.6 % [0.3–1.1]	*Longley et al.*[55]
Duration of symptoms (severe cases)	20.0 days [14.0–24.0]	*Mejia et al.*[36]
Duration of symptoms (moderate cases)	14 days [8.0–26.0]	*Mejia et al.*[36]
Disability weight (moderate typhoid)	0.051 [0.032–0.074]	*Roth et al.*[38]
Disability weight (severe typhoid)	0.133 [0.088–0.190]	*Roth et al.*[38]
Disability weight (typhoid with ileal perforation)	0.324 [0.220–0.442]	*Roth et al.*[38]
**Demographics**		
Birth Rate	Varies by setting	Bangladesh Bureau of Statistics 2021 [26]
All-cause mortality rate and life expectancy	Varies by age and setting	Bangladesh Bureau of Statistics 2021 [26]
Dhaka and non-Dhaka migration rates	Varies by age and setting	Bangladesh Bureau of Statistics 2017 [56]
Population size	Varies by age and setting	Bangladesh Bureau of Statistics 2021 [26]

All costs are in 2021 United States dollars (USD).

We obtained data on demographics and migration from the Bangladesh Bureau of Statistics [26]. We obtained data on vaccine efficacy from a phase 3 trial conducted in Bangladesh [15]. We assumed that this efficacy only applied to clinical infections; for the effect of vaccination on subclinical infections, we instead modeled reduced shedding based on data from a human challenge study [27]. Consistent with prior models of typhoid vaccination, we assumed that vaccine protection is leaky in that all members receive some benefit as opposed to some receiving all or none. We obtained data on vaccine cost from an announcement from the manufacturer [28], and cost of delivery via different routes from empirical studies [29], [30], [31], [32], [33], [34]. We obtained data on cost of illness from SEAP [35], [36], [37]. We obtained data on disability weights from the GBD [38].

### 2.4 Analyses

After calibrating the model, we used it to project outcomes for each vaccination strategy over the next 10 years. We considered three types of vaccination strategies including 1) routine vaccination through the EPI alongside the first measles vaccine dose (ages 9–12 months), 2) routine vaccination and a 1-time community catch-up campaign targeting children ages 1 to 15 years, and 3) routine vaccination and a 1-time school catch-up campaign targeting children ages 5 to 15 years with temporary vaccination upon school entry to reach those who were initially 1–4 years and missed both routine and school-based vaccination. We considered implementing these strategies nationally or only in Dhaka. In total, we evaluated the status quo (i.e., no vaccination) and 8 specific vaccination strategies.

We computed the ICERs of the strategies. We selected the optimal strategy as that with the highest ICER less than the willingness-to-pay (WTP) threshold. We used a WTP threshold of 1x the gross domestic product (GDP) per capita as is typical in cost-effective analyses for low- and middle-income countries [39].

We conducted probabilistic, one-way, and scenario sensitivity analyses. For the probabilistic sensitivity analysis, we sampled 10,000 parameter sets from distributions, ran the model, and assessed outcome distributions. For the one-way sensitivity analysis, we used linear regression metamodeling to determine the effects of individual parameters on results [40]. For the scenario sensitivity analyses, we varied assumptions including the model time period and perspective used for computing costs (i.e., societal versus healthcare) and assessed their effects on results.

## 3 Results

### 3.1 Status quo

**Fig. 2:**
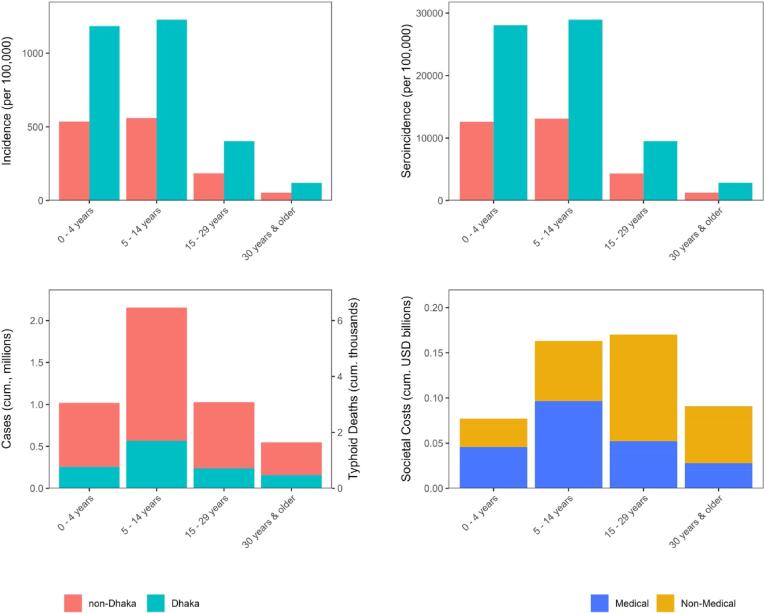
Projected typhoid health outcomes and costs over the next 10 years under the status quo. Fig. S4 shows societal costs by Dhaka and non-Dhaka residence. Abbreviations: cum., cumulative; USD, United States dollars.

Without typhoid conjugate vaccination, we projected that there will be 4.75 million clinical typhoid cases over the next 10 years in Bangladesh resulting in 14.23 thousand deaths and societal costs of $501 million (Fig. 2). Our projected clinical incidence and seroincidence by Dhaka and non-Dhaka residence and age group were consistent with our calibration targets (Fig. S1). We projected that Dhaka will have higher typhoid incidence than non-Dhaka (496.43 cases vs 248.16 cases per 100 k people). Finally, we projected that the majority of the national societal costs (55.63 %) over the next 10 years will be due to nonmedical costs such as lost wages from time spent sick.

### 3.2 Typhoid vaccination strategies

**Fig. 3:**
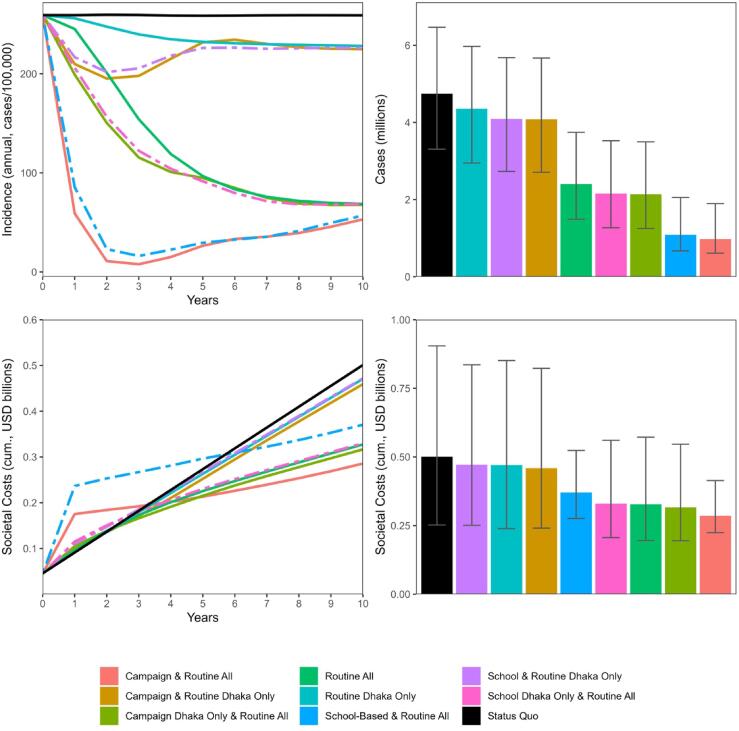
Projected typhoid health outcomes and costs over the next 10 years under various typhoid conjugate vaccination strategies. In the left panels, school-based vaccination strategies are shown by dashed lines. In the right panels, the error bars show the 2.5th and 97.5th quantiles of cases and costs across 10,000 probabilistic simulations. Abbreviations: cum., cumulative; USD, United States dollars.

We projected typhoid clinical incidence, seroincidence, and societal costs over the next 10 years for 8 vaccination strategies and the status quo (Fig. 3). All vaccination strategies substantially reduced typhoid incidence compared to the status quo. The greatest reduction was achieved with a strategy of routine immunization at 9–12 months along with catch-up campaigns in both Dhaka and non-Dhaka (3.77 million cases averted), followed by routine immunization at 9–12 months along with school-based campaigns in Dhaka and non-Dhaka (3.66 million cases averted). The strategies that only included routine vaccination were less effective than those that included routine vaccination and campaign or school-based vaccination. Although non-Dhaka has lower typhoid incidence, vaccinating this area still had substantial health benefits compared with strategies that only covered Dhaka. For example, a national program of routine immunization at 9–12 months alongside catch-up campaigns was projected to avert an additional 3.11 million cases compared to the same strategy focused only in Dhaka. We projected that the most effective strategy—campaign and routine all—would save $20 to $147 million compared to the other vaccination strategies and $172 million compared to the status quo. We projected that this strategy would have year 1 vaccination costs of $117 million and 10-year cumulative vaccination costs of $176 million.

### 3.3 Cost-effectiveness analysis

We projected the incremental costs, incremental DALYs, and ICERs associated with use of the vaccination strategies over the next 10 years (Table 2). The vaccination strategy resulting in the greatest number of DALYs averted, national routine immunization at 9–12 months with catch-up campaigns, was cost saving compared with all other vaccination strategies and the status quo.

**Table 2:** Cost-effectiveness of typhoid vaccination strategies.

**Strategy**	**Year 1 Vaccination Costs (USD Millions)**	**10-Year Cumulative Vaccination Costs (USD Millions)**	**Discounted Cumulative Costs (Societal, USD Millions)**	**Cost Savings Relative to the Status Quo (Societal, USD Millions)**	**Discounted Cumulative** **DALYs Averted Relative to the Status Quo (Thousands)**	**ICERs (Societal)**
Campaign & Routine All	117	176	221	172	275	Lowest-cost

Campaign Dhaka Only & Routine All	24	82	241	152	187	Dominated

School Dhaka Only & Routine All	33	94	255	139	186	Dominated

Routine All	7	66	251	143	166	Dominated

School-Based & Routine All	174	249	304	90	267	Dominated

Campaign & Routine Dhaka Only	17	25	359	35	50	Dominated

School & Routine Dhaka Only	26	36	371	23	49	Dominated

Routine Dhaka Only	1	8	369	25	28	Dominated

Status Quo	0	0	394	0	0	Dominated

Abbreviations: DALY, disability-adjusted life year; ICER: incremental cost-effectiveness ratio.

### 3.4 Sensitivity analyses

**Fig. 4:**
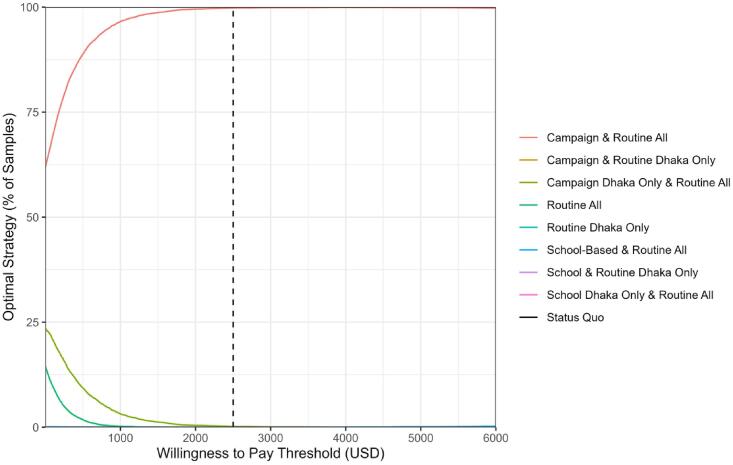
Cost-effectiveness acceptability curves. The percent of samples in which each vaccination strategy is optimal is shown across willingness to pay thresholds as computed from 10,000 probabilistic simulations. The dashed line shows the willingness to pay threshold corresponding to 1x Bangladesh GDP per capita. Abbreviations: USD, United States dollars.

In probabilistic sensitivity analysis, we found the optimal strategy, campaign and routine all, was robust to changes in parameter values and the WTP threshold (Fig. 4). Across WTP thresholds, campaign and routine all was the optimal strategy in the vast majority of samples. For example, using a 1x GDP per capita threshold, campaign and routine all was preferred in 99.78 % of simulations.

**Fig. 5:**
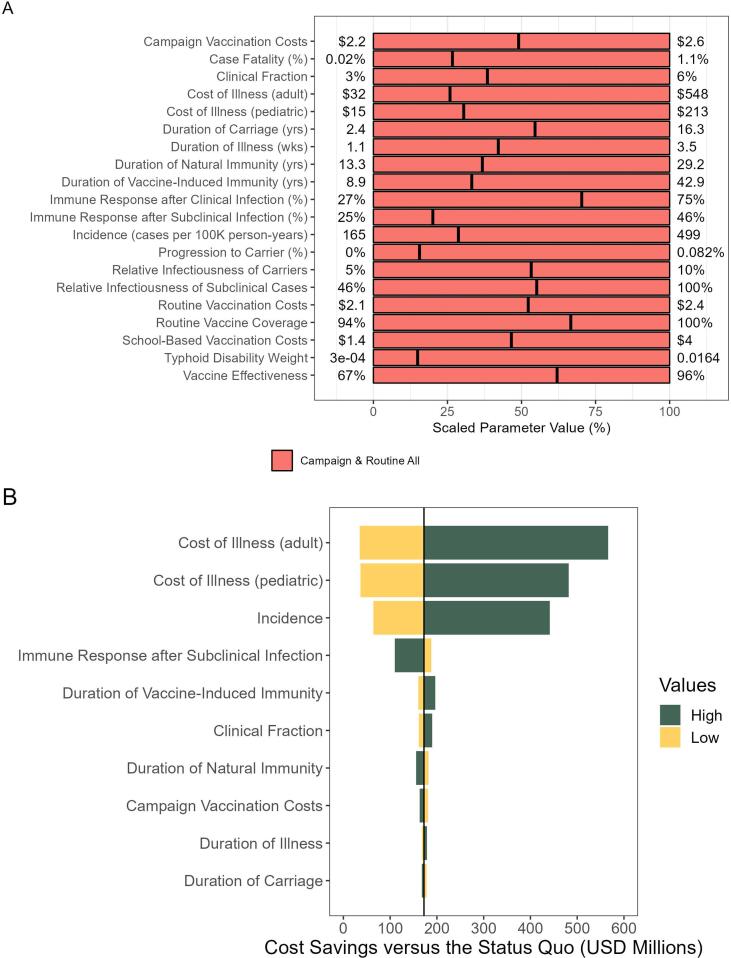
One-way sensitivity analysis. A) The optimal vaccination strategy when all parameter values are fixed at their means and one at a time is varied over its full range is shown as determined using linear regression metamodeling. The minimum and maximum values for each parameter are shown on the left and right of the figure, respectively. The x-axis shows the scaled parameter value such that 0% is the minimum, 100% is the maximum, and 50% is the median. The black lines indicate the mean value for each parameter. The color indicates which vaccination strategy is optimal. B) We also present these results as cost savings of campaign and routine all versus the status quo for the ten most influential parameters.

In one-way sensitivity analysis, we found the optimal strategy was robust to varying individual parameter values over ranges (Fig. 5). In no case did the optimal strategy change.

In scenario sensitivity analyses, we found the optimal strategy was robust to varying the model time period and perspective used for computing costs (Fig. S8). When using the healthcare perspective for costs, the optimal strategy was no longer cost saving compared to all other strategies. However, it was highly cost effective with an ICER of $489 per DALY averted and still cost saving compared to the status quo.

## 4 Discussion

We projected that a nationwide introduction of TCV at 9–12 months of age, with a catch-up campaign for children ages 1–15, would substantially reduce typhoid incidence and mortality in Bangladesh. Further, we projected this strategy would save $172 million compared to the status quo and $20 to $147 million compared to more limited TCV introduction strategies. Cost savings were driven by healthcare- and non-healthcare-associated costs of typhoid illness that would be averted through immunization. Comparing the different vaccination programs, relatively high incidence favored more aggressive approaches including vaccination in both Dhaka and non-Dhaka, and supplementing routine vaccination with catch-up vaccination. Community-based catch-up vaccination was more cost-effective than school-based catch-up vaccination due in part to potentially lower costs. The investment required to implement these strategies, however, would be substantial. We projected that a national routine and catch-up campaign strategy would require $176 million over 10 years. If such an investment is not feasible due to budget constraints, a national routine strategy with or without a catch-up campaign in Dhaka could be used instead, which we project would be less expensive but still yield substantial health benefits compared to the status quo. Our findings were robust across wide ranges of parameter values in probabilistic, one-way, and scenario sensitivity analyses.

Our findings were consistent with the previously reported epidemiology of typhoid in Bangladesh. For example, in the status quo analysis, we projected that children and Dhaka will be disproportionately affected. Our findings were also broadly consistent with past studies that have projected that typhoid conjugate vaccination can be cost effective and even cost saving in typhoid endemic areas [19], [21], [41], [42], [43], [44]. A major constraint for past country-level cost-effectiveness analyses has been a paucity of incidence data, particularly outside of selected urban settings where typhoid surveillance studies have been conducted. Incidence estimates for Pakistan, Nepal, and Bangladesh, for example, have been largely derived from studies in Karachi, Kathmandu, and Dhaka, which may not be representative of the large rural populations of each of those countries.

Recently, new sero-epidemiologic approaches have been developed to estimate typhoid incidence from cross-sectional serosurveys, which may enable cheaper and more rapid mapping of risk outside of urban areas [23], [45]. A key novelty in our model is the integration of clinical incidence data and new longitudinal serologic data during model calibration. By using Dhaka clinical incidence data in conjunction with new seroincidence data for both Dhaka and non-Dhaka, the model was effectively able to estimate clinical incidence in non-Dhaka. Thus, our policy analysis was not limited by assuming clinical incidence for Dhaka is representative of that for all of Bangladesh. Importantly, we project that even accounting for substantially lower clinical incidence and seroincidence in non-Dhaka than Dhaka, all TCV strategies would be cost saving compared to the status quo. An added benefit of using both clinical incidence and seroincidence data is that they allow for better estimation of parameters such as the fraction of clinical versus subclinical infections.

Our analysis has several limitations. First, as with all models, our model is a simplification of the complex processes of pathogen natural history and transmission, including the assumption of homogenous mixing. Second, while *S.* Typhi is transmitted by both short- and long-cycle routes, we did not explicitly model these two routes. This is common in typhoid models [21], and vaccination is not expected to differentially affect transmission via these routes. Third, there is substantial parameter uncertainty due to limited data. However, our results were robust to changes in parameter values in probabilistic, one-way, and scenario sensitivity analyses. Fourth, we only modeled Dhaka and non-Dhaka as the non-Dhaka seroincidence data were all fairly similar. Fifth, we conservatively did not model increasing drug resistance. If drug resistance increases over the model period, the costs and mortality resulting from typhoid infection would increase since treatment of infected individuals would be less effective. This would make the vaccination strategies we consider even more cost saving compared to the status quo. Insight into these scenarios can be obtained from our sensitivity analyses on the costs and mortality resulting from typhoid infection; in no sensitivity analysis were our findings regarding optimal vaccination strategy altered. Sixth, our delivery cost estimates are based on those for other vaccines due to data availability. However, there may be ways to couple TCV administration with other existing typhoid mitigation programs, such as water and sanitation programs, which could increase the cost savings of TCV programs compared to our projections. Nevertheless, our results were robust to changes in delivery costs in our sensitivity analyses. Seventh, we estimated substantial indirect effects of approximately ∼ 80 % by comparing the average rate of susceptible to clinically infected under the status quo and campaign and routine all over the same time period as for a cluster randomized trial in Bangladesh [15]. The cluster randomized trial did not find significant indirect effects. This difference might be because we assumed a much higher vaccine coverage, because there could have been spread between the clusters in the trial, or because our model overestimated vaccine impact on shedding and transmission. Finally, we did not consider paratyphoid fever in our analysis as there are currently no paratyphoid vaccines. Paratyphoid fever, however, accounts for ∼ 12 % of enteric fever cases in Bangladesh, and this proportion may grow when TCVs are introduced [17].

In summary, we project that nationwide TCV introduction would substantially reduce typhoid incidence and would very likely be cost saving in Bangladesh. Such a strategy could have an immediate impact on disease burden, at a time when antimicrobial resistance is a growing threat. However, consistent with previous models and a recent cluster-randomized trial in Dhaka, the projected typhoid incidence in Dhaka would remain “high” (>100 per 100,000) under all vaccine introduction scenarios, highlighting the need for investments and innovation in improving water and sanitation alongside vaccine introduction.

## 5 Authorship criteria

All authors attest they meet the ICMJE criteria for authorship.

## Funding

This work was supported by the Bill and Melinda Gates Foundation [grant numbers INV-000572, INV-008335].

## CRediT authorship contribution statement

**Christopher Weyant:** Writing – review & editing, Writing – original draft, Visualization, Validation, Software, Resources, Project administration, Methodology, Investigation, Formal analysis, Data curation, Conceptualization. **Yogesh Hooda:** Writing – review & editing, Visualization, Validation, Supervision, Resources, Project administration, Methodology, Investigation, Funding acquisition, Formal analysis, Data curation, Conceptualization. **Sira Jam Munira:** Writing – review & editing, Visualization, Validation, Supervision, Resources, Project administration, Methodology, Investigation, Funding acquisition, Formal analysis, Data curation, Conceptualization. **Nathan C. Lo:** Writing – review & editing, Visualization, Validation, Resources, Project administration, Methodology, Investigation, Funding acquisition, Formal analysis, Data curation, Conceptualization. **Theresa Ryckman:** Writing – review & editing, Visualization, Validation, Resources, Project administration, Methodology, Investigation, Funding acquisition, Formal analysis, Data curation, Conceptualization. **Arif M. Tanmoy:** Writing – review & editing, Visualization, Validation, Supervision, Resources, Project administration, Methodology, Investigation, Funding acquisition, Formal analysis, Data curation, Conceptualization. **Naito Kanon:** Writing – review & editing, Visualization, Validation, Supervision, Resources, Project administration, Methodology, Investigation, Funding acquisition, Formal analysis, Data curation, Conceptualization. **Jessica C. Seidman:** Writing – review & editing, Visualization, Validation, Supervision, Resources, Project administration, Methodology, Investigation, Funding acquisition, Formal analysis, Data curation, Conceptualization. **Denise Garrett:** Writing – review & editing, Visualization, Validation, Supervision, Resources, Project administration, Methodology, Investigation, Funding acquisition, Formal analysis, Data curation, Conceptualization. **Samir K. Saha:** Writing – review & editing, Visualization, Validation, Supervision, Resources, Project administration, Methodology, Investigation, Funding acquisition, Formal analysis, Data curation, Conceptualization. **Jeremy D. Goldhaber-Fiebert:** Writing – review & editing, Visualization, Validation, Supervision, Resources, Project administration, Methodology, Investigation, Funding acquisition, Formal analysis, Data curation, Conceptualization. **Senjuti Saha:** Writing – review & editing, Visualization, Validation, Supervision, Resources, Project administration, Methodology, Investigation, Funding acquisition, Formal analysis, Data curation, Conceptualization. **Jason R. Andrews:** Writing – review & editing, Visualization, Validation, Supervision, Resources, Project administration, Methodology, Investigation, Funding acquisition, Formal analysis, Data curation, Conceptualization.

## Declaration of competing interest

The authors declare that they have no known competing financial interests or personal relationships that could have appeared to influence the work reported in this paper. NCL reports consulting fees from the World Health Organization related to guidelines on neglected tropical diseases, which are outside the scope of the present work.

## Data Availability

Data will be made available on request.
