# Supplementary material for: Cost-effectiveness and public health impact of typhoid conjugate vaccine introduction strategies in Bangladesh
Source: Vaccine. 2024 Apr 19;42(11):2867–76. doi: 10.1016/j.vaccine.2024.03.035 (PMC11033679; doi:10.1016/j.vaccine.2024.03.035)
Supplement: Supplementary data 1 [file mmc1.docx]

**Cost-effectiveness and public health impact of typhoid conjugate vaccine**

**introduction strategies in Bangladesh**

**Supplement**

This supplement provides additional details on model calibration, projections, cost-effectiveness analyses, and sensitivity analyses.

**Figure S1A.** Projected Dhaka clinical cases from calibrated model versus calibration targets.

**
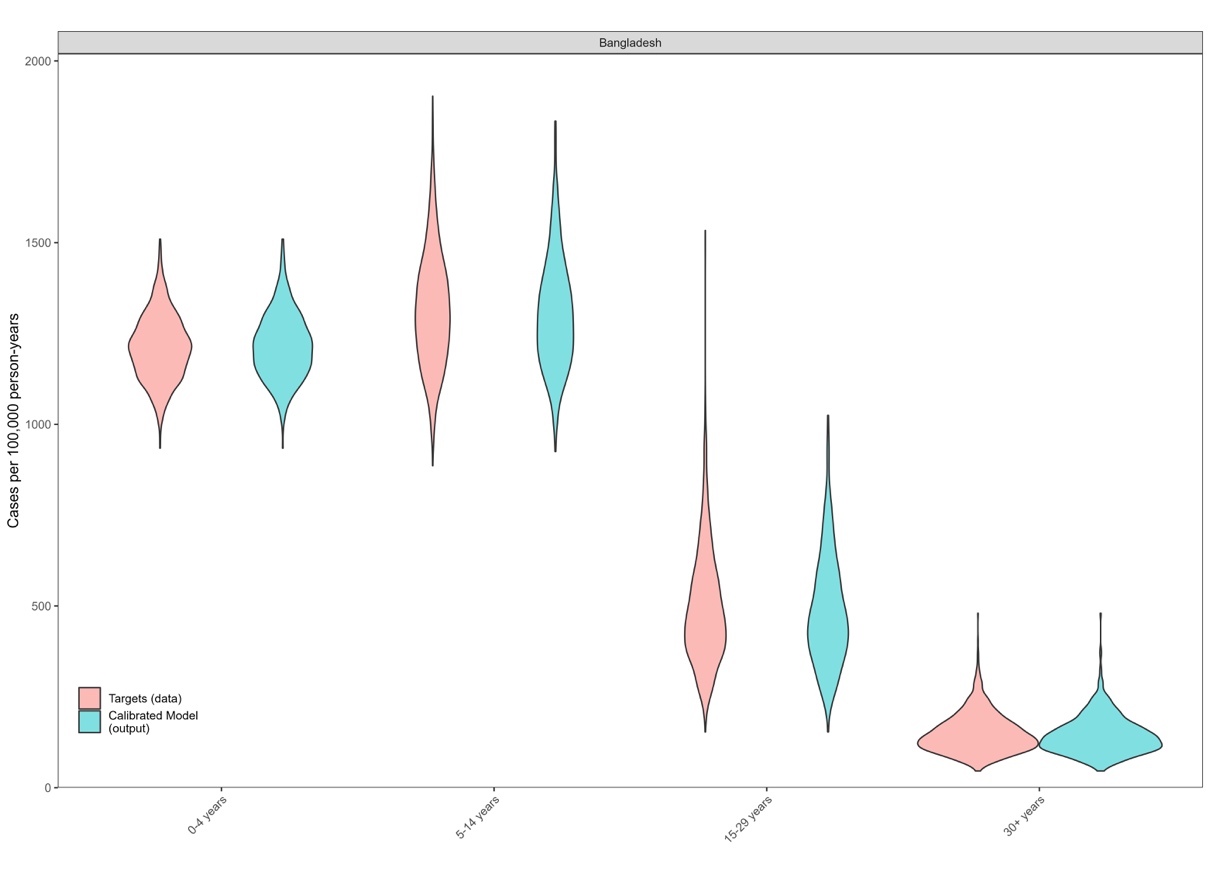
**

**Figure S1B.** Projected Dhaka serological cases from calibrated model versus calibration targets.


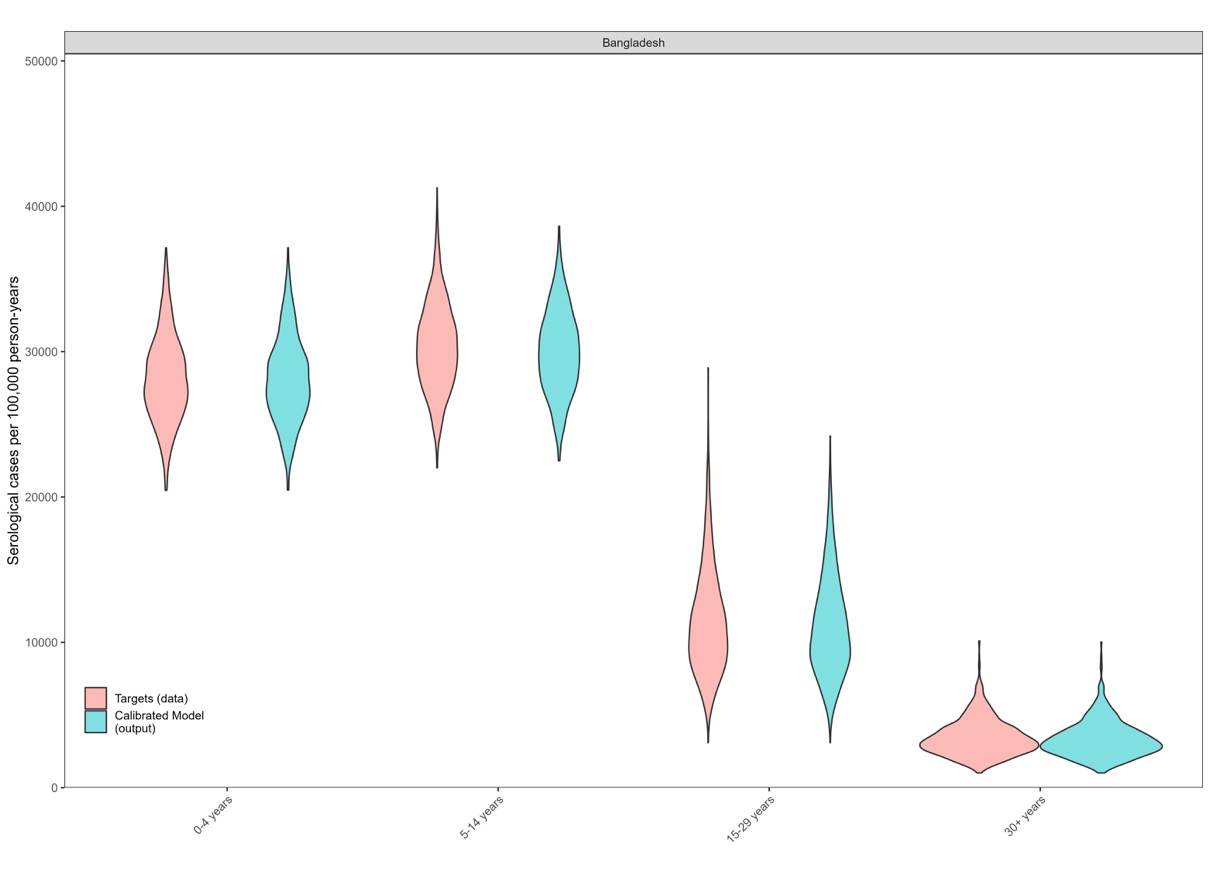


**Figure S1C.** Projected non-Dhaka serological cases from calibrated model versus calibration targets.


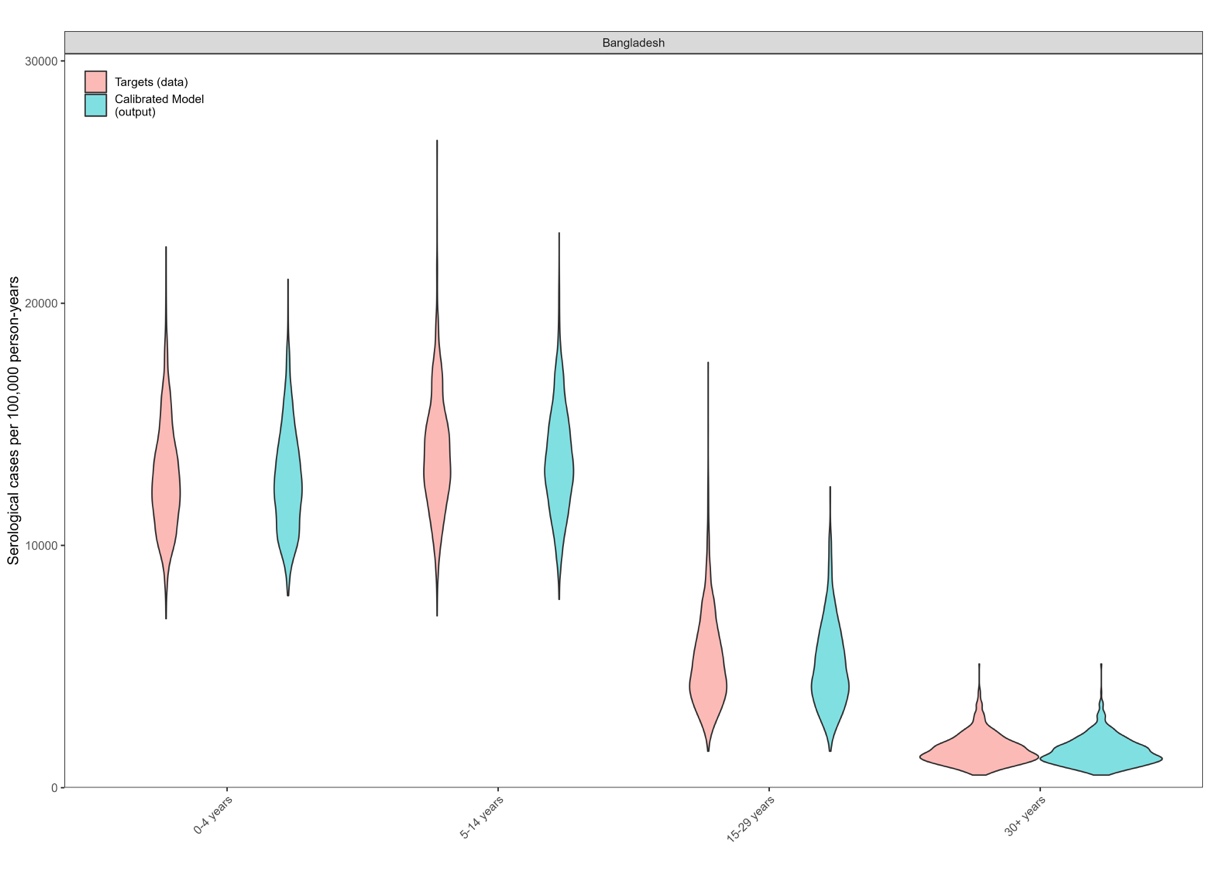


**Figure S2.** Calibrated Dhaka parameter distributions. Histograms of the 10,000 parameter sets.


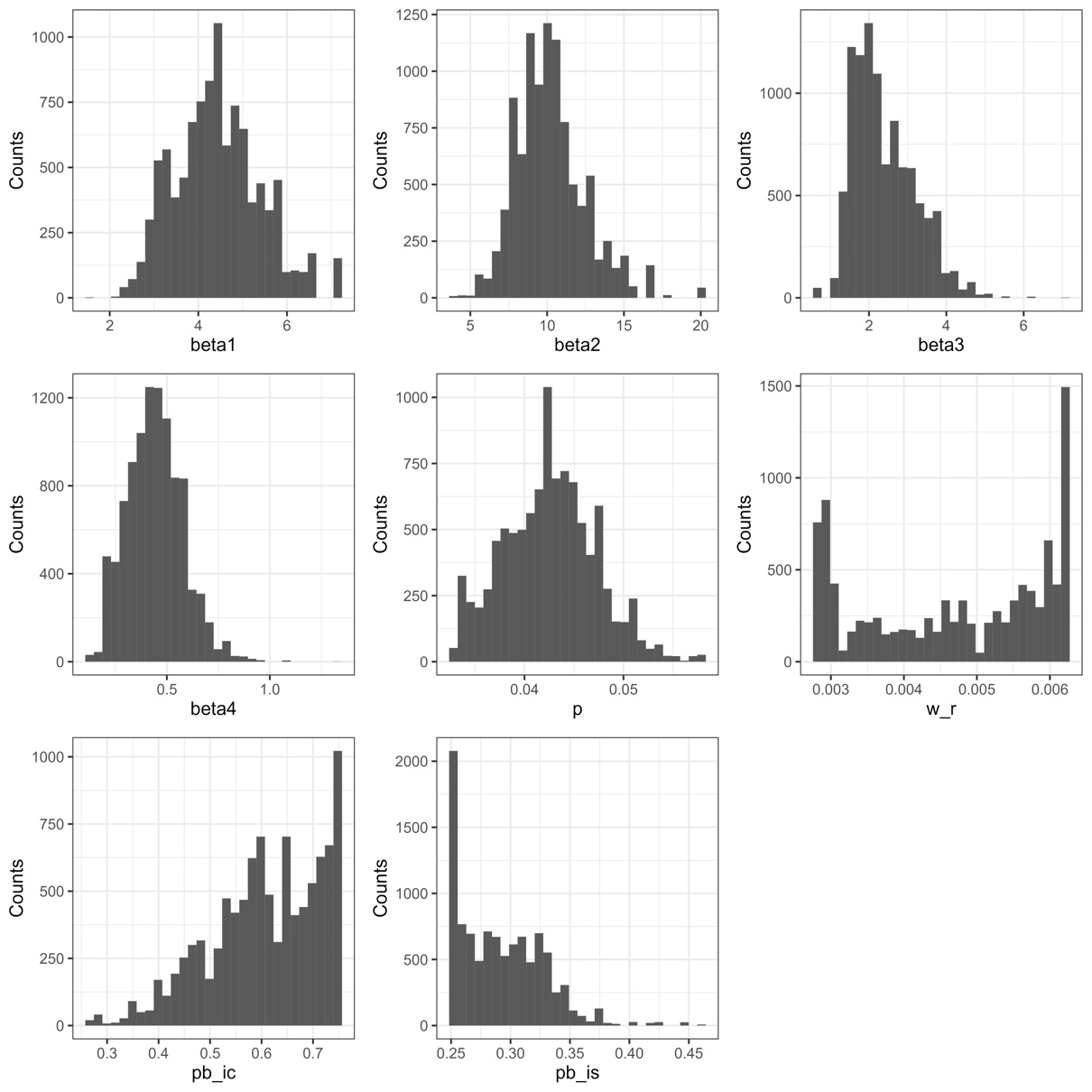


Abbreviations: p, fraction clinical versus subclinical infections; w_r, rate of loss of immunity; pb_ic, fraction immune response after clinical infection; pb_is, fraction immune response after subclinical infection. In the parameter names, the numbers 1 to 4 denote the age groups 0-4, 5-14, 15-29, and 30+ years, respectively.

**Figure S3.** Calibrated non-Dhaka parameter distributions. Histograms of the 10,000 parameter sets.


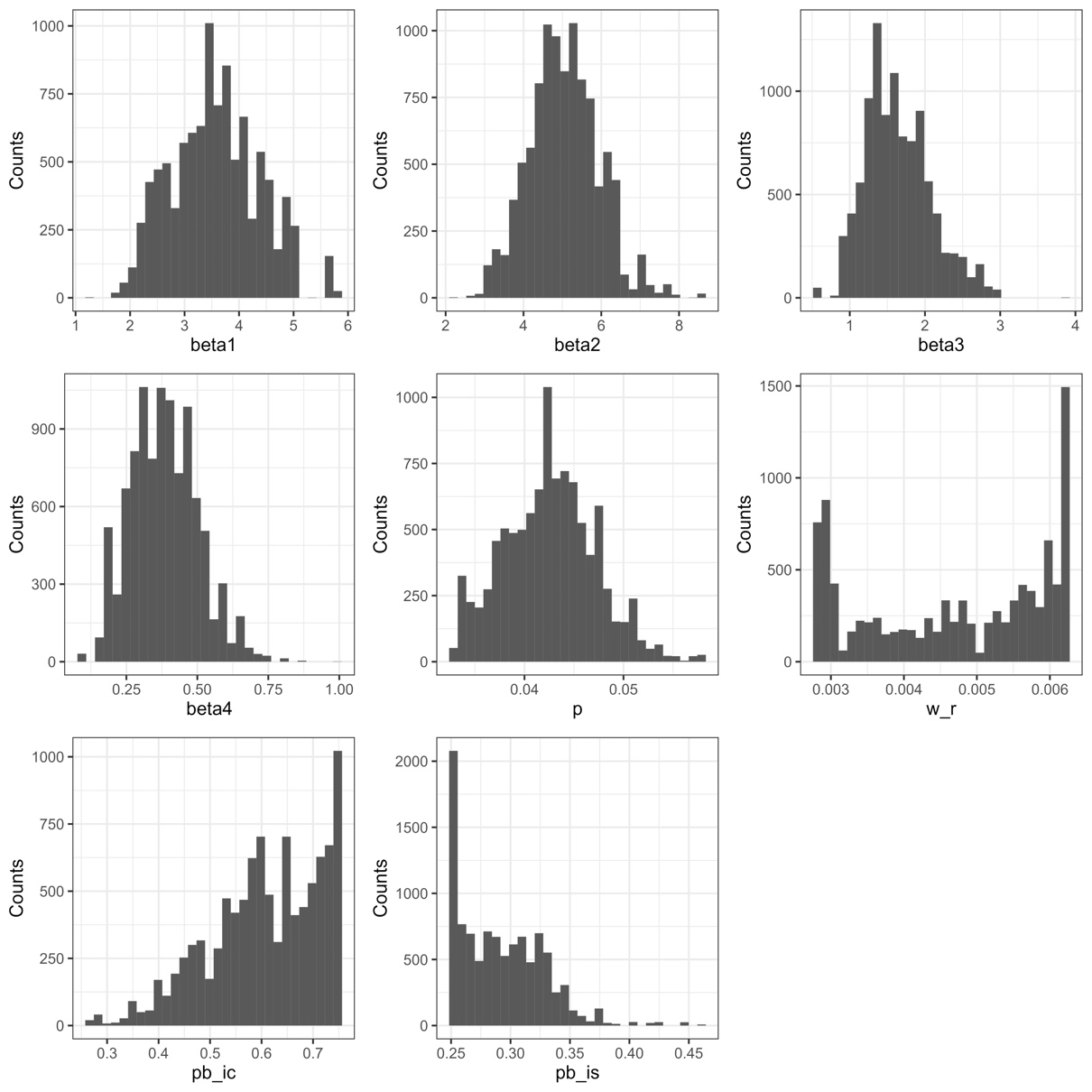


Abbreviations: p, fraction clinical versus subclinical infections; w_r, rate of loss of immunity; pb_ic, fraction immune response after clinical infection; pb_is, fraction immune response after subclinical infection. In the parameter names, the numbers 1 to 4 denote the age groups 0-4, 5-14, 15-29, and 30+ years, respectively.

**Figure S4.** Status quo cost projections by age and perspective.


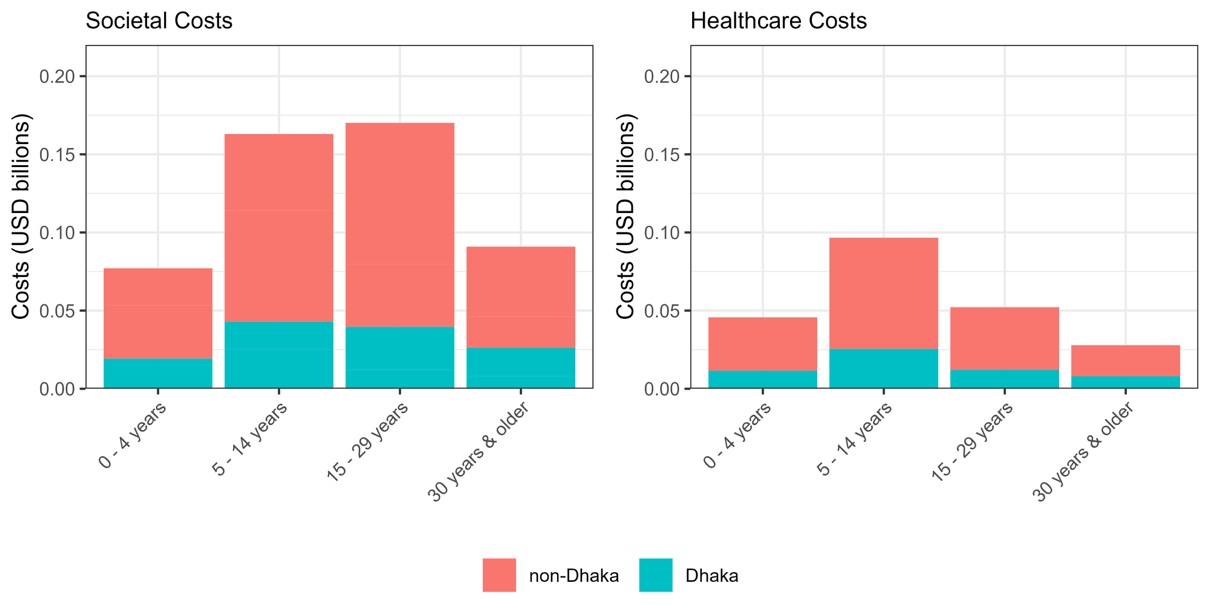


**Figure S5.** Cost projections by perspective.


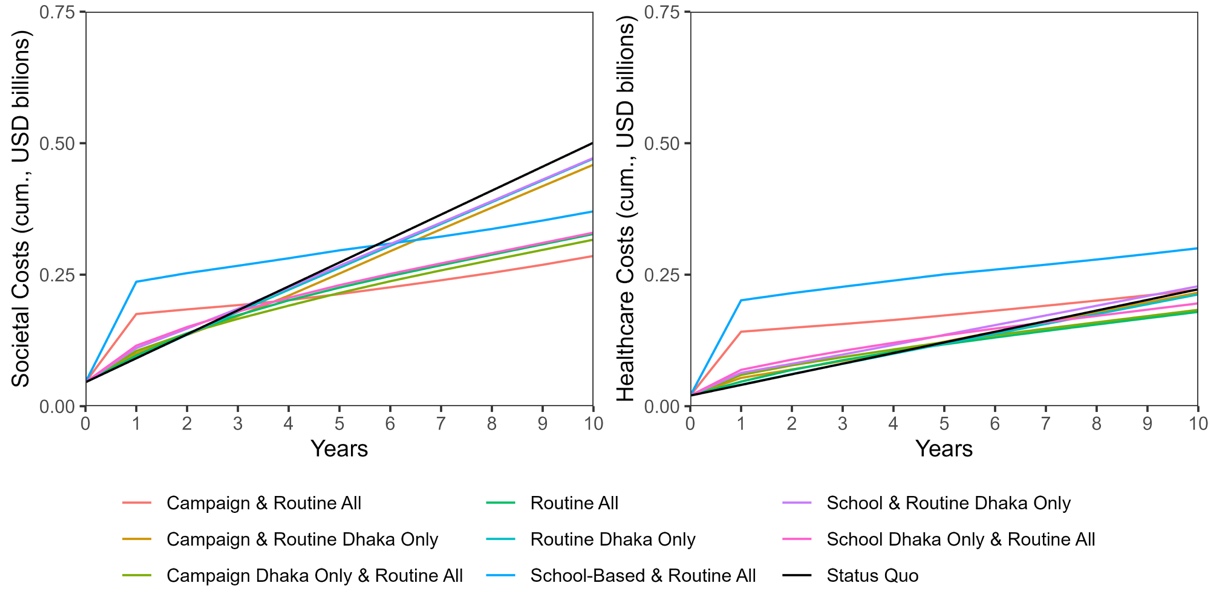


**Figure S6.** Start-up vaccination cost projections. The year 1 healthcare vaccination costs are shown by strategy.


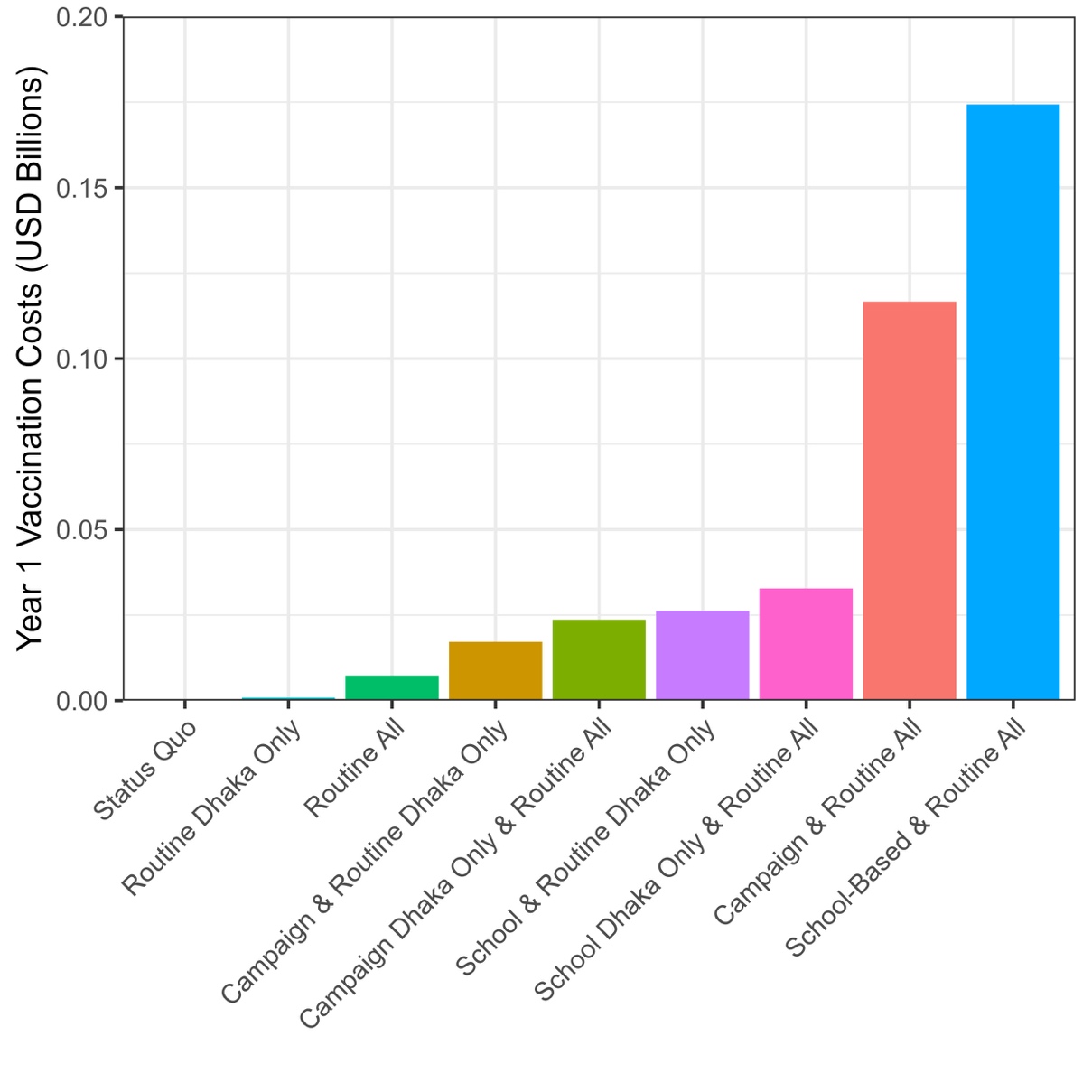


**Figure S7.** Case projections by age and setting.

**
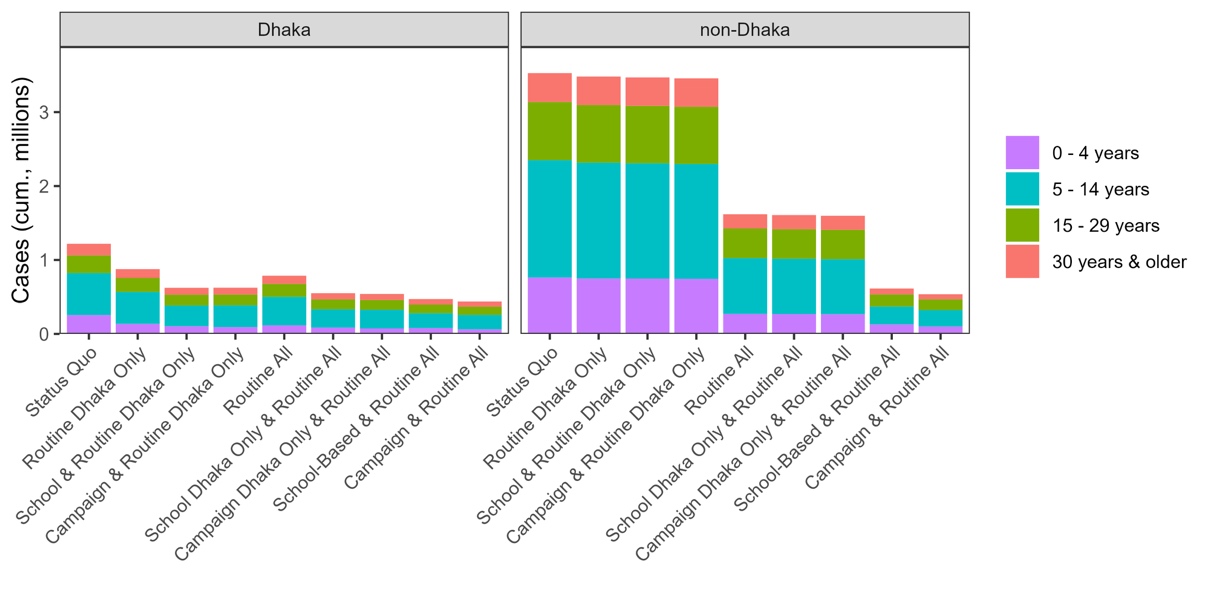
**

**Figure S8.** Cost-effectiveness projections by time horizon and perspective.

**
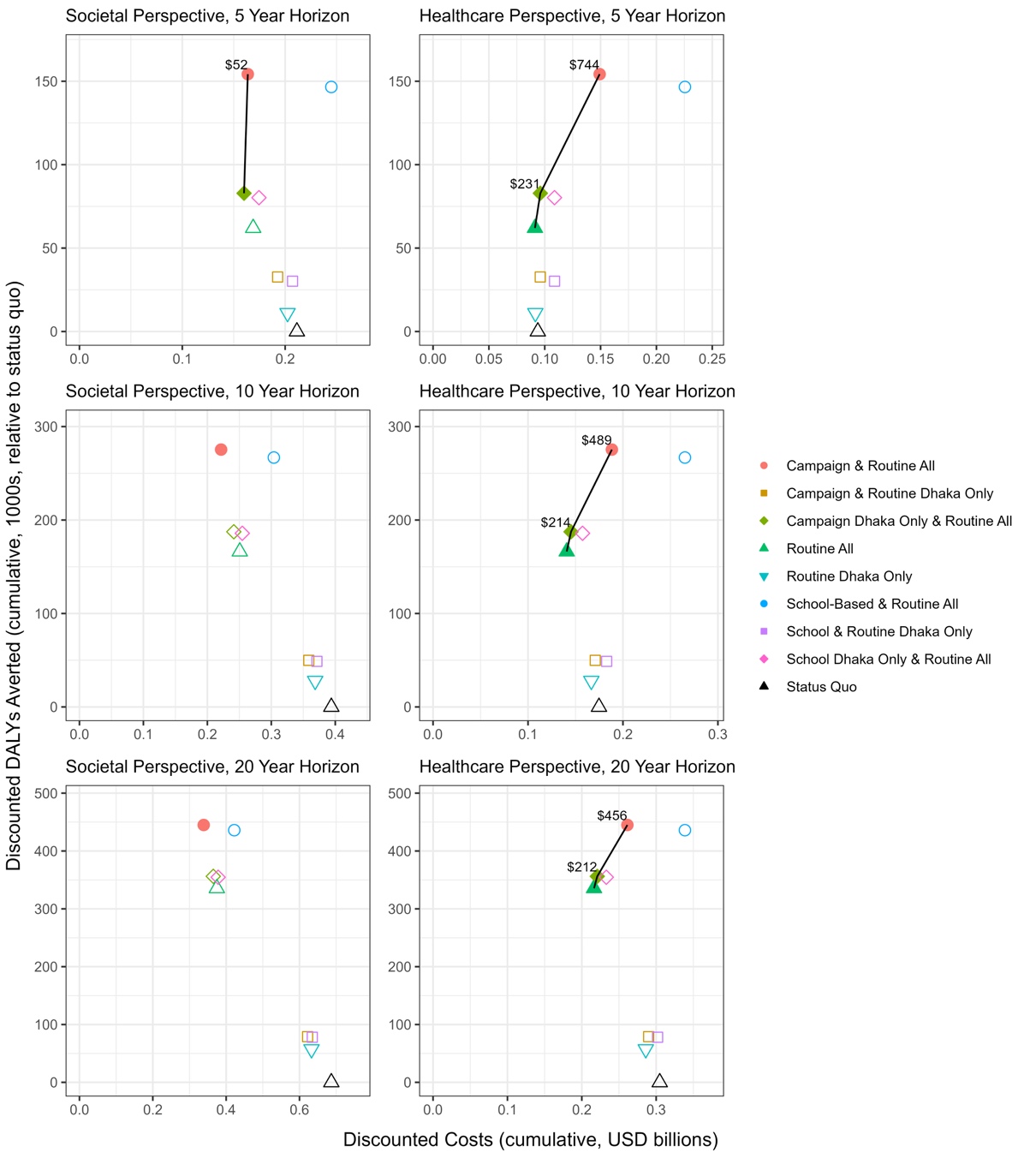
**

**Figure S9.** Cost-effectiveness acceptability frontier.

**
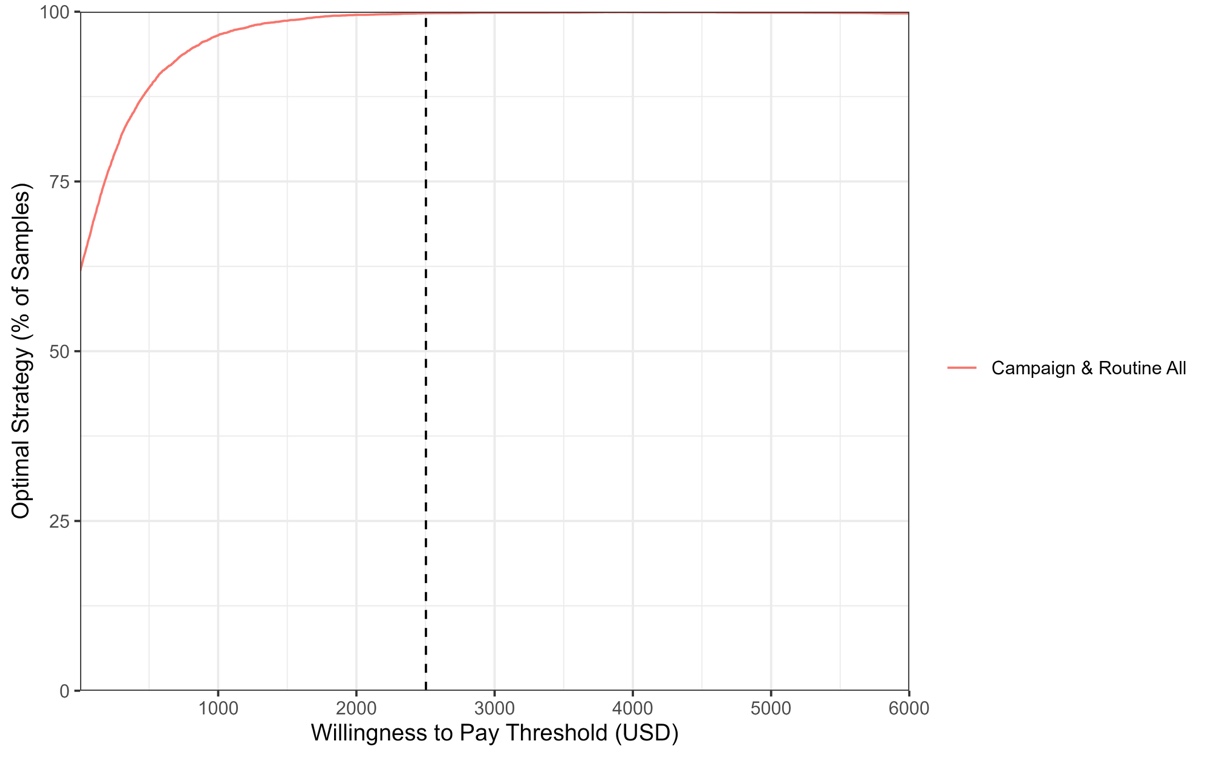
**

**Figure S10.** Projections of the effects of routine vaccination timing on health outcomes and costs.

**
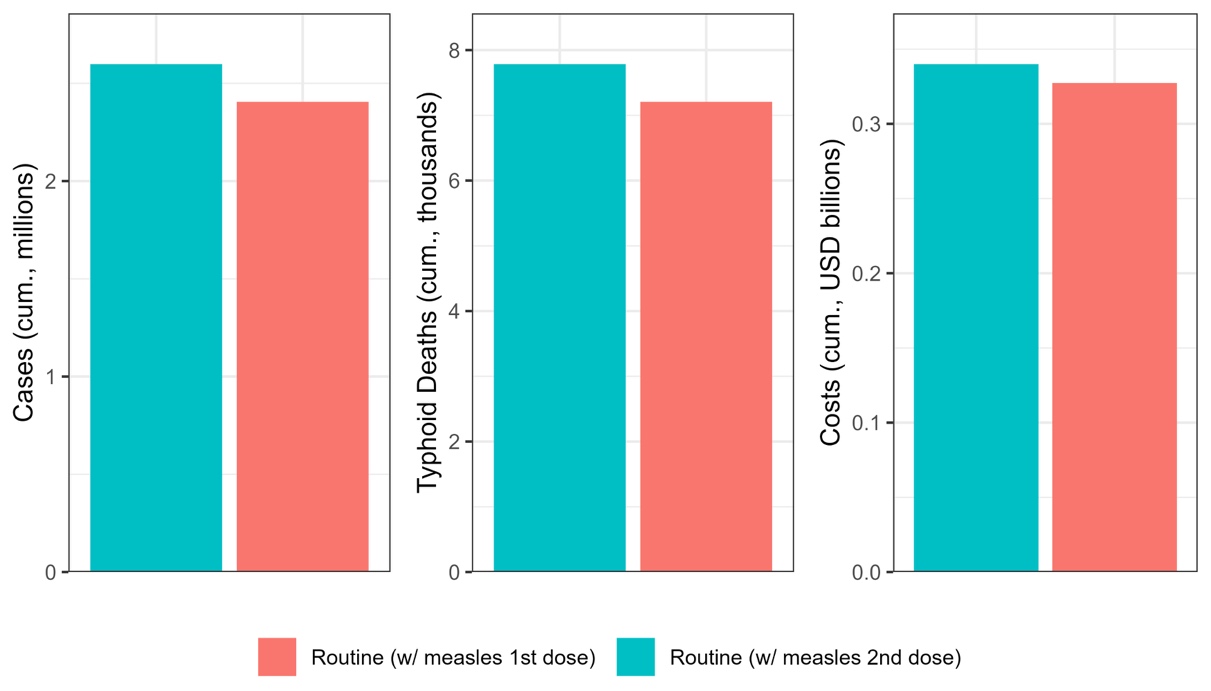
**

**Figure S11.** Cost-effectiveness acceptability curve and frontier from the healthcare perspective.

**
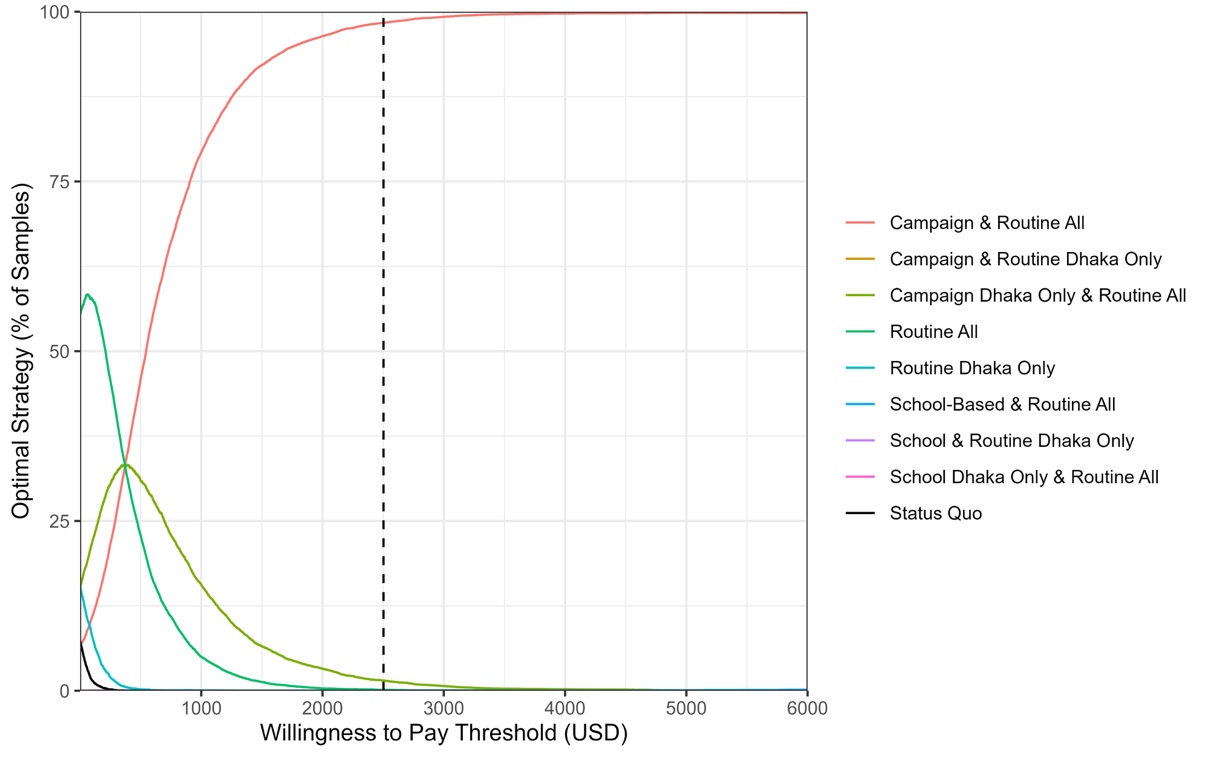
**

**
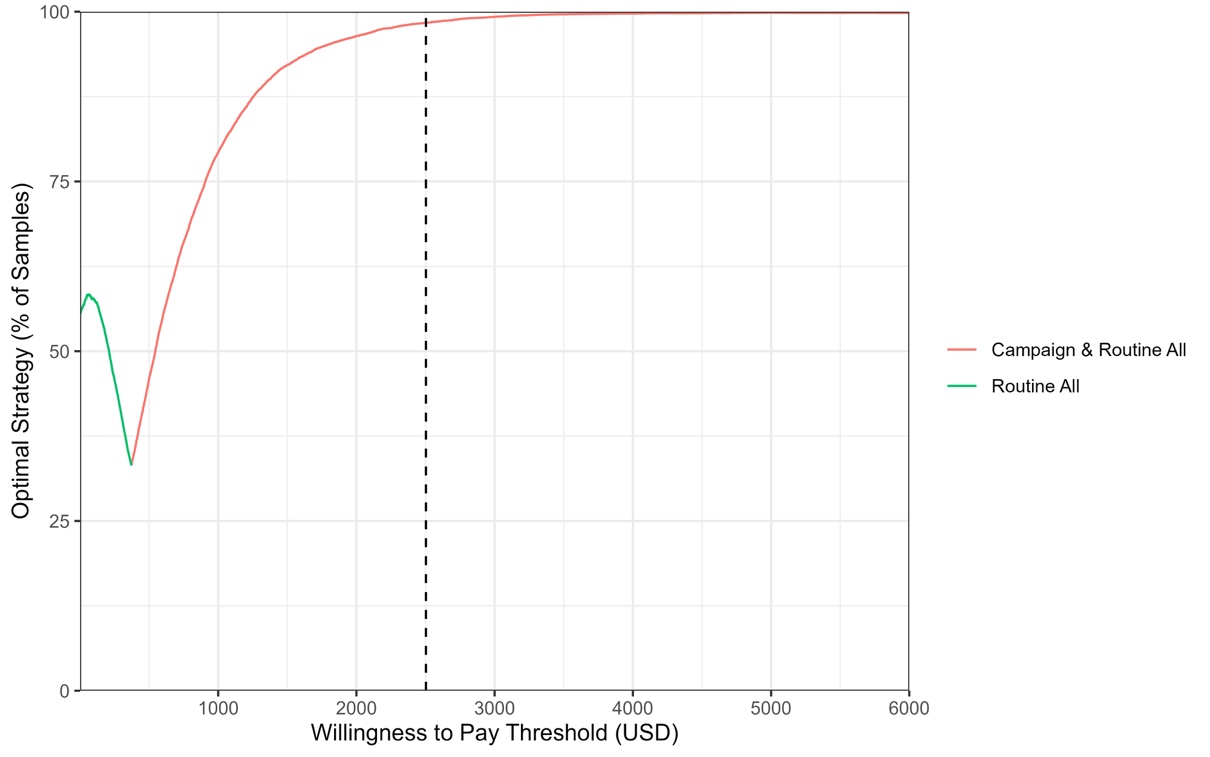
**

**Figure S12.** One-way sensitivity analysis from the healthcare perspective.


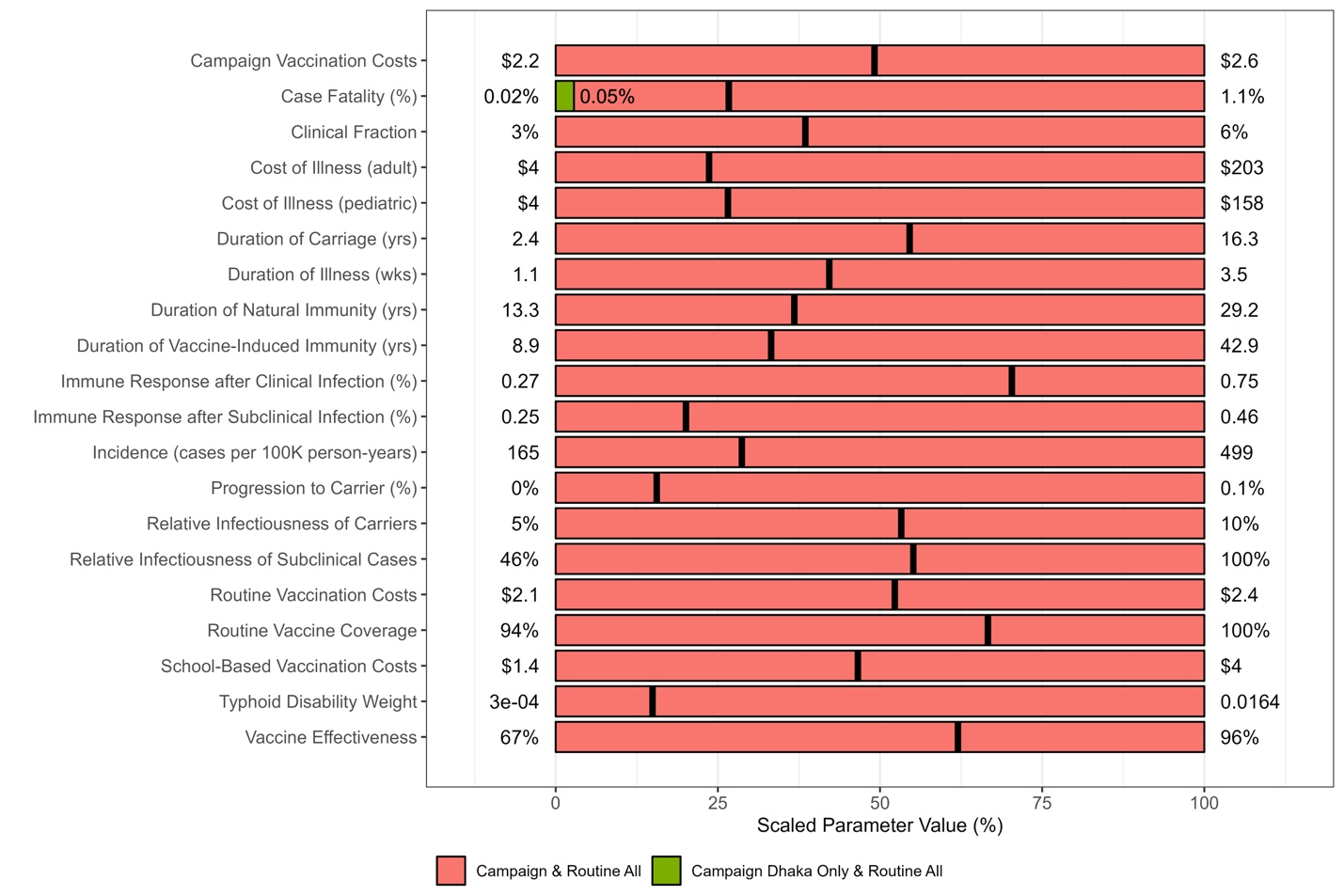


**Text S1.** Detailed model description.

We extended an existing mathematical model of typhoid transmission. The model is a dynamic transmission compartmental model, and the population is stratified by typhoid status, Dhaka and non-Dhaka residence, and age group (17 age groups). Typhoid transmission is modeled as a series of ordinary differential equations, which are coded using the *DifferentialEquations* package in Julia. For each location (i.e., Dhaka or non-Dhaka) *i* and age *j*, there are 7 equations with one for each typhoid status. These equations are described below. We model monthly time steps. People are assumed to interact with other people in the same location; we also model aging and migration, but they are not shown in the equations for simplicity.

The change in the susceptible population ($S$) over time ($t$) is given by Equation 1. People enter into the susceptible population via births (rate $u_{B_{i,j}}$), loss of immunity from recovered (R) at rate $\omega_{r}$, loss of immunity from vaccinated ($V$) at rate $\omega_{v}$, and not mounting an immune response following from both clinically infected ($I_{c}$) and subclinically infected ($I_{s}$) with probability $\left( 1-b \right)$ and rate $\gamma$. People leave the susceptible population via new infections, vaccination, and deaths. For new infections, they occur based on the rate $\beta$, which is calibrated and varies by location and four broad age groups, and the number of infected people including clinically infected $I_{C}$, subclinically infected $I_{S}$, carriers $C$, and subclinically infected while vaccinated $V_{S}$. Subclinical infections and carriers are less infectious than clinical infections with a relative risk of transmission $r_{\mathrm{Is}}$ for subclinical infections, $r_{c}$ for carriers, and $r_{\mathrm{Vs}}$ for subclinical infections while vaccinated. Campaign vaccination occurs at rate ${vx}_{i,j}$and depends on the vaccination strategy being modeled (e.g., it is zero for the status quo). Routine vaccination occurs with some probability when people age out of the 0 to <9 months age category as it is assumed to occur with the first measles dose given at 9 months. Background mortality from the susceptible population and all other populations occurs at rate $\mu_{i,j}$.

[1: Susceptible] ${{dS}_{i,j}}/{dt}=u_{B_{i,j}}-\beta_{i,j}S_{i,j}I_{C_{i,j}}-\beta_{i,j}S_{i,j}I_{S_{i,j}}r_{\mathrm{Is}}-\beta_{i,j}S_{i,j}C_{i,j}r_{c}+\beta_{i,j}S_{i,j}V_{S_{i,j}}r_{Vs}{+\omega}_{r}R_{i,j}+\omega_{v}V_{i,j}+\left( 1-b \right)(1-\theta_{j})\gamma(I_{c_{i,j}}+I_{s_{i,j}})-{vx}_{i,j}S_{i,j}-\mu_{i,j}S_{i,j}$

The change in the clinically infected ($I_{C}$) and subclinically infected ($I_{S}$) populations are given by Equations 2 and 3. People enter these populations via new infections from the susceptible population as described above. Of these, a proportion $p$ enter the clinically infected population while $\left( 1-p \right)$ enter the subclinically infected population. People can also enter the clinically infected population from the vaccinated population via new infections, which occur at a much lower transmission rate based on vaccine efficacy $\alpha$. Vaccinated people who become subclinically infected enter their own population ($V_{S}$) as described below. Vaccinated people who become subclinically infected do so at the same rate as unvaccinated people; however, they are less infectious based on the relative risk of transmission $r_{\mathrm{Vs}}$. People recover and leave the infected populations at a rate $\gamma$. People in the clinically infected population face an elevated risk of mortality $\mu_{Ic}$ in addition to background mortality. People leave the subclinically infected population by being vaccinated. We assume that this population may be vaccinated as subclinically infected people are difficult to distinguish from susceptible people in practice.

[2: Clinically Infected] ${dI_{C_{i,j}}}/{dt}=p\left[ \beta_{i,j}S_{i,j}I_{C_{i,j}}+\beta_{i,j}S_{i,j}I_{S_{i,j}}r_{\mathrm{Is}}+\beta_{i,j}S_{i,j}C_{i,j}r_{C}+\beta_{i,j}S_{i,j}V_{S_{i,j}}r_{Vs} \right]+\left( 1-\alpha\right)p\left[ \beta_{i,j}V_{i,j}I_{C_{i,j}}+\beta_{i,j}V_{i,j}I_{S_{i,j}}r_{\mathrm{Is}}+\beta_{i,j}V_{i,j}C_{i,j}r_{C}+\beta_{i,j}V_{i,j}V_{S_{i,j}}r_{Vs} \right]-\gamma I_{C_{i,j}}-\left( \mu_{i,j}+\mu_{Ic} \right)I_{C_{i,j}}$

[3: Subclinically Infected] ${dI_{S_{i,j}}}/{dt}=\left( 1-p \right)\left[ \beta_{i,j}S_{i,j}I_{C_{i,j}}+\beta_{i,j}S_{i,j}I_{S_{i,j}}r_{Is}+\beta_{i,j}S_{i,j}C_{i,j}r_{C}+\beta_{i,j}S_{i,j}V_{S_{i,j}}r_{Vs} \right]-\gamma I_{S_{i,j}}-{vx}_{i,j}I_{S_{i,j}}-\mu_{i,j}I_{S_{i,j}}$

The change in the carrier population ($C)$ is given by Equation 4. People enter this population via infected people becoming carriers upon recovery, which occurs with an age-specific probability $\theta_{j}$. Carriers continue to be infectious but at a lower rate. People leave the carrier population to the recovered population, which occurs at rate $\omega_{c}$.

[4: Carriers] ${{dC}_{i,j}}/{dt}=\gamma\theta_{j}(I_{C_{i,j}}+I_{S_{i,j}}+V_{S_{i,j}})-\omega_{c}C_{i,j}-{\mu_{i,j}C}_{i,j}$

The change in the recovered population ($R)$ is given by Equation 5. People enter this population from the infected populations and carrier population as previously described. People leave this population as they lose immunity and return to the susceptible population, which occurs at rate $\omega_{r}$.

[5:Recovered/Immune]${{dR}_{i,j}}/{dt}=b\gamma(1-\theta_{j})(I_{C_{i,j}}+{I_{S}}_{i,j})+{\gamma(1-\theta_{j}){V_{S}}_{i,j}+\omega}_{c}C_{i,j}-\omega_{r}R_{i,j}- \mu_{i,j}R_{i,j}$

The change in the vaccinated population ($V$) is given by Equation 6. People enter this population from the susceptible population via vaccination at rate ${vx}_{i,j}$. People leave this population as they are clinically or subclinically infected as previously described. People also leave this population as they lose immunity, which occurs at rate $\omega_{v}$.People in all states except clinically infected can be vaccinated as they are hard to distinguish from susceptible people; however, only susceptible people experience a meaningful change in immunity and thus are shown in the equation below. People in these other states being vaccinated will still contribute to other outcomes such as costs.

[6:Vaccinated] ${{dV}_{i,j}}/{dt}={vx}_{i,j}S_{i,j}-\left( 1-\alpha\right)$p$\left[ \beta_{i,j}V_{i,j}I_{C_{i,j}}+\beta_{i,j}V_{i,j}I_{S_{i,j}}r_{Is}+\beta_{i,j}V_{i,j}C_{i,j}r_{C}+\beta_{i,j}V_{i,j}V_{S_{i,j}}r_{Vs} \right]-\left( 1-p \right)\left[ \beta_{i,j}V_{i,j}I_{C_{i,j}}+\beta_{i,j}V_{i,j}I_{S_{i,j}}r_{Is}+\beta_{i,j}V_{i,j}C_{i,j}r_{C}+\beta_{i,j}V_{i,j}V_{S_{i,j}}r_{Vs} \right]-\omega_{v}V_{i,j}-\mu_{i,j}V_{i,j}$

The change in the vaccinated population with subclinical infection ($V_{S})$is given by Equation 7. People enter this population as vaccinated people are subclinically infected as previously described. People also enter this population as subclinically infected people are vaccinated. People leave this population as they recover as previously described.

[7: Vaccinated and Subclinically Infected] ${{dV}_{S_{i,j}}}/{dt}=\left( 1-p \right)\left[ \beta_{i,j}V_{i,j}I_{C_{i,j}}+\beta_{i,j}V_{i,j}I_{S_{i,j}}r_{Is}+\beta_{i,j}V_{i,j}C_{i,j}r_{C}+\beta_{i,j}V_{i,j}V_{S_{i,j}}r_{Vs} \right]-\gamma V_{S_{i,j}}+{vx}_{i,j}I_{S_{i,j}}-\mu_{i,j}V_{S_{i,j}}$

**Text S2.** Additional information on serosurveys.

Table S1 shows the communities where the serosurveys were conducted and the population densities of these communities. Table S2 shows information about the tests conducted in each community including the number of children tested, mean and median ages of these children, and time periods over which tests were conducted. Roughly 400 children were tested per community. For Dhaka and Mirzapur, households with children from the SEAP healthcare utilization study were randomly preselected for potential enrollment. If the preselected household was not enrolled, then a nearest neighbor approach was used to identify an alternate household. See Aiemjoy *et al.* for additional details [1]. For Chittagong, Dinajpur, Satkhira, and Sylhet, two schools were selected per community that were intended to be representative of that community based on the expert opinion of our Bangladeshi coauthors. Students from these schools were enrolled using convenience sampling. For Faridpur, the same procedure was used to enroll students except that four schools were selected instead of two. The serosurveys were only conducted in children as further work is needed to extend the method to adults, which is more challenging due to factors such as potentially increased chance of multiple exposures and differential waning [1]. As we only had seroincidence targets for children, we assumed seroincidence follows the same age distribution as that for clinical incidence, which allowed us to generate seroincidence targets for the older age groups. Serosurveys were conducted and seroincidences were estimated for each community using published methods [1].

**Table S1.** Serosurveys were performed in 7 communities that included urban, semi-urban, and rural areas. Population densities were estimated from circles with 5 km radii drawn around each study site. For Dhaka, a polygon for Mirpur was used instead since that was where the study was conducted.

| **Site location** | **Population density (people/km^2^)** |
| --- | --- |
| Dhaka | 71,347 |
| Faridpur | 2,926 |
| Chittagong | 2,075 |
| Sylhet | 1,849 |
| Mirzapur | 1,451 |
| Satkhira | 857 |
| Dinajpur | 782 |

**Table S2.** Serosurveys were performed in 7 communities and roughly 400 children were tested in each community.

| **Site location** | **Number of children tested** | **Mean age** | **Median age** | **Testing start date** | **Testing end date** |
| --- | --- | --- | --- | --- | --- |
| Dhaka | 401 | 9.4 | 9.2 | 2019-06-30 | 2021-01-12 |
| Faridpur | 395 | 10.2 | 9.0 | 2022-01-12 | 2022-01-13 |
| Chittagong | 395 | 10.6 | 10.0 | 2022-06-21 | 2022-06-22 |
| Sylhet | 389 | 13.2 | 14.0 | 2022-06-13 | 2022-06-15 |
| Mirzapur | 596 | 9.7 | 9.9 | 2019-07-17 | 2021-06-19 |
| Satkhira | 400 | 12.5 | 13.0 | 2022-05-29 | 2022-05-31 |
| Dinajpur | 393 | 11.0 | 11.0 | 2022-04-17 | 2022-04-19 |

**References**

[1] Aiemjoy K, Seidman JC, Saha S, Munira SJ, Sajib MSI, Sium SMA, et al. Estimating typhoid incidence from community-based serosurveys: a multicohort study. The Lancet Microbe 2022;3.
